# Reversible complete left bundle branch block and a wide QRS complex following administration of sodium-glucose cotransporter-2 inhibitor and volume reduction in a patient with systolic heart failure: a case report

**DOI:** 10.1093/ehjcr/ytae512

**Published:** 2024-09-14

**Authors:** Masaki Takenaka, Satoshi Yanagisawa, Yukihiko Yoshida, Yasuya Inden, Toyoaki Murohara

**Affiliations:** Department of Cardiology, Takabari Heart Clinic, 1-1525 Takabari, Meito-ku, Nagoya, Aichi 465-0061, Japan; Department of Cardiology, Nagoya University Graduate School of Medicine, 65 Tsurumai, Showa, Nagoya, Aichi 466-8550, Japan; Department of Advanced Cardiovascular Therapeutics, Nagoya University Graduate School of Medicine, 65 Tsurumai, Showa, Nagoya, Aichi 466-8550, Japan; Department of Cardiology, Japanese Red Cross Aichi Medical Center Nagoya Daini Hospital, 2-9 Myoken-cho, Showa, Nagoya, Aichi 466-8650, Japan; Department of Cardiology, Nagoya University Graduate School of Medicine, 65 Tsurumai, Showa, Nagoya, Aichi 466-8550, Japan; Department of Cardiology, Nagoya University Graduate School of Medicine, 65 Tsurumai, Showa, Nagoya, Aichi 466-8550, Japan

**Keywords:** Complete left bundle branch block, QRS duration, Cardiac resynchronization therapy, Heart failure, Sodium-glucose cotransporter-2 inhibitors, Case report

## Abstract

**Background:**

Guidelines recommend optimal medical therapy before cardiac resynchronization therapy (CRT) implantation. Herein, we report the potential effect of sodium-glucose cotransporter-2 inhibitors (SGLT2is) in improving the QRS duration and volume reduction in a patient with complete left bundle branch block (CLBBB) and reduced cardiac function.

**Case summary:**

A 68-year-old man with a history of ischaemic cardiomyopathy and decreased cardiac function had exacerbation of heart failure (HF) at an outpatient clinic. His QRS duration increased remarkably with a CLBBB of 143 ms on an electrocardiogram, and left ventricular desynchrony was assessed by echocardiography, suggesting an indication of CRT implantation. Administration of an SGLT2i and multimodal treatment for HF stabilized his HF condition and improved the QRS duration and volume reduction thereafter. The CLBBB recovered to incomplete LBBB with a QRS duration of 112 ms on electrocardiography after 6 months. The patient has been stably followed up with continuous medications, including SGLT2i, without requiring CRT implantation or worsening of HF for 12 months.

**Discussion:**

This case presents a unique scenario wherein electrical and mechanical reverse remodelling occurred in a patient with systolic HF and CLBBB, highlighting the potential benefits of SGLT2i in HF management. It may be important to carefully consider CRT indications when seeking other options to treat HF conditions and recognize an unusual phenomenon of reverse LBBB in clinical cases.

**Learning points**

- Sodium-glucose cotransporter-2 inhibitor (SGLT2i) administration to treat heart failure (HF) can potentially improve the QRS duration and CLBBB, resulting in electrical and mechanical reverse remodelling in patients with HF.
- The potential benefits of an SGLT2i in HF management may extend beyond its recognized effects on outcomes and mortality.
- It is crucial to learn the importance of considering alternative treatment options and optimizing medical therapy before cardiac resynchronization therapy device implantation.

## Introduction

Pathophysiology of heart failure (HF) comprises several mechanisms involving complex inter-organ interactions. Heart failure can also affect the conduction system. Gap junction remodelling and interstitial fibrosis have been linked to impaired electrical conduction velocity in HF.^1^ Here, we report a case of recovery of a wide QRS complex of complete left bundle branch block (CLBBB) after HF management and a possible benefit of administering sodium-glucose cotransporter-2 inhibitors (SGLT2is) beyond their effects on HF outcomes.

## Summary figure

**Figure:**
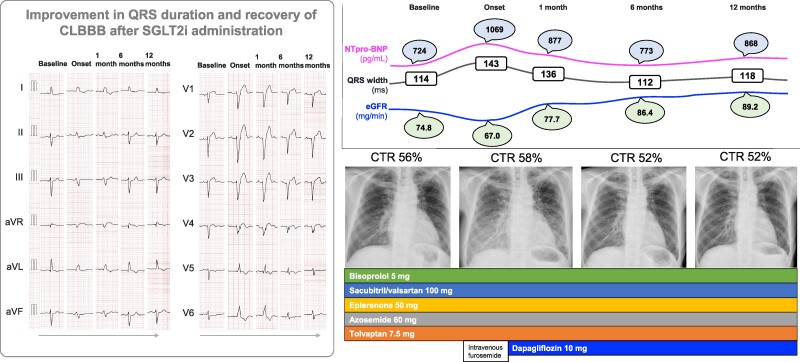


## Case presentation

A 68-year-old man with recently developed shortness of breath and slight leg oedema visited an outpatient clinic. The patient had been treated earlier for myocardial infarction and HF with reduced left ventricular ejection fraction (LVEF) and was in a stable condition for the past 7 years. At the time of examination, his blood pressure, pulse rate, and oxygen saturation level were 92/64 mmHg, 102 b.p.m., and 92% while breathing room air, respectively.

The patient had undergone treatment for acute anterior wall myocardial infarction 13 years prior with emergency percutaneous coronary intervention and coronary artery bypass surgery for residual coronary ischaemic lesions within the same year. Subsequently, the patient continued to receive the standard medications for HF: bisoprolol (5 mg), sacubitril/valsartan (100 mg), eplerenone (50 mg), tolvaptan (7.5 mg), and azosemide (60 mg) at the outpatient clinic and had been hospitalized for HF once 7 years ago. After discharge, echocardiography revealed an LVEF of 41.7% and a cardiothoracic ratio of 52% on chest radiography. The N-terminal pro-brain natriuretic peptide (NT-proBNP) level was 1380 pg/mL, and the New York Heart Association (NYHA) function was Class III.

**Figure 1:**
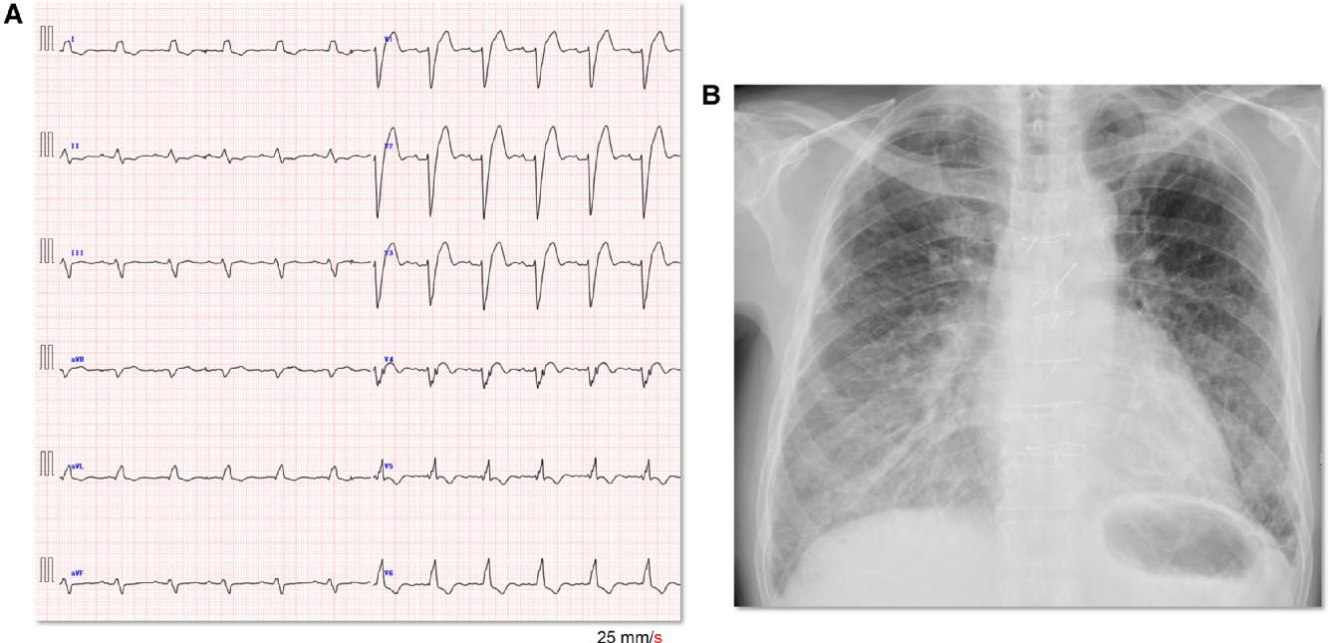
Examination results at the time of heart failure exacerbation. A 12-lead electrocardiogram showing a complete left bundle branch block and QRS duration of 143 ms (*A*). The heart rate was 74 b.p.m. Cardiomegaly with an increased cardiothoracic ratio of 58% and enhanced pulmonary congestion on chest radiography (*B*).

At the examination, electrocardiography showed a CLBBB with a QRS width of 143 ms (*Figure 1A*), which had increased significantly from 114 ms 7 years ago. Chest radiography revealed an increased cardiothoracic ratio of 58% with enhanced pulmonary congestion and cephalization (*Figure 1B*). Transthoracic echocardiography using a modified Simpson’s method demonstrated severe akinesis in the anteroseptal wall and apex, with a decreased LVEF of 38%. The left ventricular end-systolic volume (LVESV) and end-diastolic volume (LVEDV) were 120 and 178 mL, respectively. Left ventricular dyssynchrony, which was not observed before exacerbation, was also observed (see Supplementary material online, *Videos S1* and *S2*). Blood examination revealed increased NT-proBNP levels of 1069 pg/mL and impaired renal function with an estimated glomerular filtration rate (eGFR) of 67.0 mL/min. Accordingly, the patient was classified as having NHYA functional Class IV.

The patient declined admission and requested continuous treatment at the outpatient clinic. Therefore, he was prescribed a daily treatment schedule of intravenous loop diuretic therapy and additionally administered an SGLT2i, dapagliflozin (10 mg). All other medications were continued without modification.

**Figure 2:**
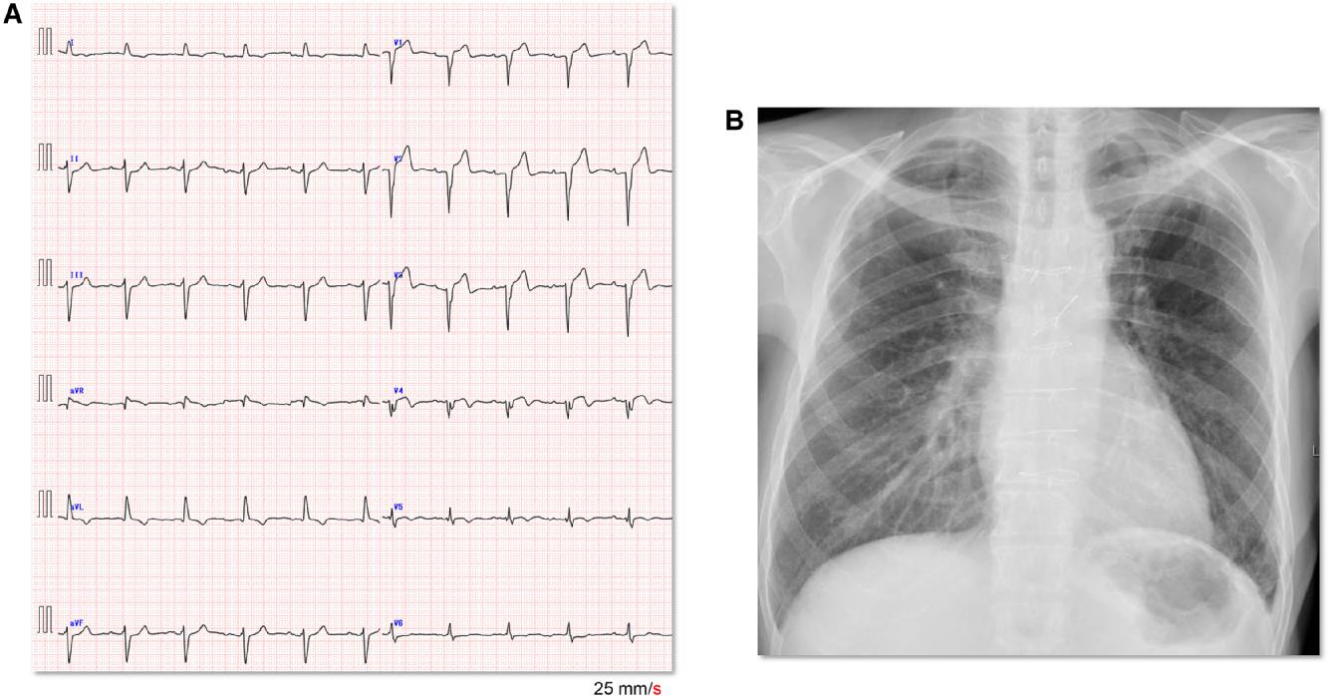
Examination results after 6 months of treatment. The QRS duration decreased by 112 ms on electrocardiography (*A*). The heart rate was 65 b.p.m. Improved pulmonary congestion and cardiothoracic ratio by 52%, as shown by radiography (*B*).

During the acute phase, 3 days of intermittent infusion of intravenous furosemide (20 mg/day) improved the patient’s condition. Presuming that the unstable condition was a part of the recuperative process following diuretic infusion and that the HF volume management was controllable, we opted to add only dapagliflozin 10 mg to the patient’s medications at the outpatient clinic and followed up. When the HF worsened, the troponin-T level was measured at 0.012 ng/mL. An additional adenosine-administered stress thallium-201 scintigraphy revealed no evidence of new or worsened ischaemia. One month later, NTpro-BNP levels quickly decreased to 877 pg/dL, and the QRS duration on the ECG shortened from 143 to 136 ms. Six months after administering the aforementioned medications, the QRS duration improved to 112 ms (*Figure 2*), resulting in an incomplete LBBB. Simultaneously, the NT pro-BNP level decreased to 773 pg/dL, eGFR recovered to 86.4 mg/min, and NYHA function improved to Class II status. Thus, we continued medical treatment in the outpatient clinic while the indication for cardiac resynchronization therapy (CRT) implantation settled based on a QRS duration of <130 ms with the recommendation of the latest guidelines.^2^

After 12 months, the QRS duration was 118 ms, and the NT pro-BNP level and eGFR were 868 pg/dL and 89.2 mg/min, respectively. A follow-up echocardiography after 8 months demonstrated a decreased LVEF of 24.7%, although with continuous improvements in the LVESV and LVEDV of 117.8 and 155.5 mL, respectively; a further follow-up echocardiography at 12 months revealed a markedly improved LVEF of 35% with the LVESV and LVEDV of 106 and 161.9 mL, respectively, and synchronized left ventricular contraction maintenance (see Supplementary material online, *Video S3*).

## Discussion

Typically, CLBBB is caused by disturbances in electrical conduction and is considered an irreversible impairment. However, in some cases, the CLBBB recovered with the narrowing of the QRS complex through an improvement in the HF condition. A few studies have reported this specific phenomenon based on the possible explanations: changes in the cardiac conduction system with impulse transmission, intuitively improved ventricular function, and decreased ventricular volumes.^3–5^ Moreover, decreased wall stress in a patient with ischaemic heart disease could have decreased myocardial oxygen demand and contributed to LBBB resolution.^6^ Such an atypical CLBBB can be resolved by medical therapy and volume reduction, possibly leading to an improvement in conduction delay and shortening of QRS duration.^3–5^

In the present case, the only change in medication was the addition of an SGLT2i. Indeed, a 3-day intravenous diuretic infusion could have helped reduce the flooded volume and improve HF management. However, even after cessation of diuretic infusion, the QRS duration and NT-proBNP levels continued to improve during follow-up. Sodium-glucose cotransporter-2 inhibitor is a recent breakthrough drug for improving prognoses in patients with decreased LVEF with a Class I indication to treat HF.^7,8^ We hypothesize that SGLT2i might have an additional underlying effect on reverse remodelling of electrical and mechanical disorders to improve conduction disturbance and prevent fibrosis development in addition to volume control, possibly resulting in a further shortening of the QRS duration.^9^ Nonetheless, LVEF improvement after SGLT2i administration was reported to be not relevant in contrast to ventricular volume reduction.^10^ Although a reason for the temporal decrease in LVEF after 8 months is unknown, an activated sympathetic nervous tone at the acute phase or delayed improvement in the LVESV compared with that in LVEDV might have caused the fluctuating course of LVEF in this case. Furthermore, although the additional effect of an SGLT2i on QRS duration remains to be elucidated,^11,12^ improved QRS duration, such as in the present case, may be possible in a few select individuals.

This case study also presents a cautionary note regarding the early implantation of CRT. Caution is advised about examining true CRT indications when seeking other therapies to treat HF conditions when uncertain. Administration of optimal medication before CRT implantation is a crucial issue strongly addressed in recent guidelines.^2^ Ischaemic cardiomyopathy is known to be less likely to respond to CRT than non-ischaemic HF.^13^ In the current study, baseline LVEF of 38% was above 35%, which is not a true indication for CRT,^2^ although apparent hyper-contraction status might have existed owing to the acute exacerbation, needing a repeated evaluation of cardiac function thereafter. The treatment options should be based on balancing the benefits and risks for individual patients. However, this patient may have an alternative indication of implantable cardioverter-defibrillator (ICD) implantation owing to a low LVEF (≤35%) and ischaemic cardiomyopathy.^14^ The borderline value of LVEF 35%, in this case, requires a careful follow-up of cardiac function recovery and prompt decision of prophylaxis ICD treatment, if applicable.

## Supplementary Material

ytae512_Supplementary_Data

## Data Availability

The data underlying this article will be shared upon reasonable request to the corresponding author.

## Lead author biography

Masaki Takenaka is a specialist in the field of electrophysiology and cardiac rehabilitation. He received his PhD in prognosis of the response to cardiac resynchronization therapy using myocardia viability and dyssynchrony parameters from the Nagoya University. He has been active in the area of arrhythmia, cardiology, general internal medicine, and cardiac rehabilitation at the Takabari Heart Clinic, Nagoya, Japan.

## Supplementary material

**Consent:** The authors confirm that written consent for submission and publication of this case report, including images and associated text, has been obtained from the patient in line with the COPE guidelines.

**Funding:** None declared.
